# Genetic variations in medical research in the past, at present and in the future

**DOI:** 10.2183/pjab.97.018

**Published:** 2021-06-11

**Authors:** Yoichiro KAMATANI, Yusuke NAKAMURA

**Affiliations:** *1 Laboratory of Complex Trait Genomics, Graduate School of Frontier Sciences, The University of Tokyo, Tokyo, Japan.; *2 Cancer Precision Medicine Center, Japanese Foundation for Cancer Research, Tokyo, Japan.

**Keywords:** genetic variations, reverse genetics, biobank, genome wide association study (GWAS), pharmacogenomics, immunogenomics

## Abstract

As we look so different, our genomic sequences vary enormously. The differences in our genome, genetic variations, have played very significant roles in medical research and have contributed to improvement of medical managements in the last 2–3 decades. Genetic variations include germline variations, somatic mutations, and diversities in receptor genes of rearranged immune cells, T cells and B cells. Germline variants are in some cases causative of genetic diseases, are associated with the risk of various diseases, and also affect drug efficacies or adverse events. Some somatic mutations are causative of tumor development. Recent DNA sequencing technologies allow us to perform single-cell analysis or detailed repertoire analysis of B and T cells. It is critically important to investigate temporal changes in immune environment in various anatomical regions in the next one to two decades. In this review article, we would like to introduce the roles of genetic variations in medical fields in the past, at present and in the future.

## Introduction

We have known for many years that some diseases are genetically determined, and the susceptibility to most of diseases or our phenotypes such as height or blood types are heritable. Genetic variations used/applied in genetic and medical researches are classified into five classes, RFLP (restriction fragment length polymorphism), VNTR (variable number of tandem repeat), STR (short tandem repeat or microsatellite), SNP (single nucleotide polymorphism), and CNV (copy number variation), and other SVs (structural variations) have also been reported.

## 1 Genetic variations

History of studies in genetic variations is not so long. The first DNA-based genetic variation was reported by Kan and Dozy in 1978.^1)^ They found a polymorphism of DNA sequence adjacent to human β-globin gene. Until the PCR (polymerase chain reaction) method was established, DNA polymorphisms were detected by a combination of DNA restriction enzymes and Southern blotting method. When genetic variations are present in DNA sequences recognized by any restriction endonuclease, the lengths of DNA fragments digested with these enzymes become different individual by individual and can be visualized by Southern blotting and subsequent hybridization with ^32^P-labeled DNA fragments. However, most of restriction fragment length polymorphism (RFLP) markers were bi-allelic and their heterozygosity was not so high as 10–50%. Before the development of PCR, RFLP was detected by Southern analysis and required a large amount (about 5 µg) of non-degraded genomic DNAs. In 1985, Jeffreys *et al.* reported our genome has the “minisatellite” regions that are highly polymorphic and were used as DNA fingerprinting markers for forensic purpose such as individual identification and paternity/maternity tests.^2)^ Since these minisatellite markers were difficult to examine chromosomal segregation, we attempted to isolate single-locus variable number of tandem repeat (VNTR) markers and reported in 1987.^3)^ The first VNTR marker was reported by Wyman and White in 1980,^4)^ but systematic VNTR screening was done after the discovery of “minisatellite” loci. Genetic linkage maps with VNTR markers and hundreds of RFLP markers reported by White and his colleagues made it possible to perform reverse genetics approaches for genetic diseases of unknown causes or with no biomarkers. VNTRs markers were also applied in forensic studies^5)^ and used in the clinic to monitor transplanted cells in recipients having bone marrow transplants.^6)^

VNTRs also contributed to discovery of loci of tumor suppressor genes; both alleles of the tumor suppressor genes were inactivated (the two-hit mutation model) and in many cases the inactivation of one allele occurs as the loss of a whole or a part of chromosome (loss of heterozygosity; LOH) including a tumor suppressor gene. The first systematic analysis of loss of all chromosomal arms in colorectal cancer tissues using a set of VNTR markers was performed by Vogelstein and his colleagues.^7)^ The results led to establish the multi-step carcinogenesis model of human cancers^8)^ and subsequent analysis of a short arm of chromosome 17 proved the p53 gene as a tumor suppressor gene,^9)^ which is now shown to be mutated in nearly half of all human cancers.

## 2 Reverse genetics

The usefulness of RFLP markers in medical research was first suggested by Botstein and colleagues in 1980.^10)^ RFLP and VNTR show co-dominant Mendelian inheritance and can distinguish the parental alleles (a maternal or paternal origin) of the particular chromosomal loci in our genome. These markers made reverse genetics possible by examining co-segregation of each polymorphic “marker” allele with the inheritance of any genetic diseases with Mendelian inheritance. For example, Gusella *et al.* identified the genetic locus for Huntington disease to chromosome 4.^11)^ The development of widely-available DNA polymorphic markers in late 1980s, genetic loci responsible for genetic diseases including cystic fibrosis,^12)^ multiple endocrine neoplasia type 1 (MEN1),^13)^ Friedreich’s ataxia,^14)^ ataxia telangiectasia,^15)^ familial polyposis coli,^16)^ neurofibromatosis type 1,^17)^ and familial breast cancer,^18,19)^ had been discovered and subsequently their responsible genes were identified although it took a few to several years from the mapping of the disease loci to the discovery of responsible genes. In fact, we identified the locus for familial polyposis coli to a long arm of chromosome 5 in 1987^16)^ and isolated its responsible APC (adenomatous polyposis coli) gene in 1991.^20)^ These reverse genetics approach proved that when DNAs from the family members are available, the linkage analysis with polymorphic markers is a very powerful tool to discover responsible genes for genetic diseases in any inheritance models even if no knowledge for the biological or biochemical mechanisms (or abnormalities) is obtained.

## 3 PCR and microsatellite markers

The development of polymerase chain reaction (PCR) method coupled with isolation of thermostable DNA polymerase revolutionized the speed of DNA analysis. After 1990, scientists introduced microsatellite analysis based on the PCR technology. Microsatellite markers, which are repetitive DNA segments of two to several base-pairs,^21)^ show high levels of heterozygosity and are present at >100,000 regions covering most of our genome. Because it requires a very small amount (∼10 ng) of DNAs and is applicable to the high-throughput PCR system, microsatellite analysis accelerated linkage analysis or population genetics. Furthermore, even if DNAs are degraded at some extent (for example, DNAs from FFPE samples), microsatellite loci can be still analyzed.

One of the advantages of the approach using microsatellite markers is that they can be applied to homozygosity mapping of recessive diseases with very low incidence^22)^: for example, when patients are born by marriage of relatives or individuals living in the same area for a long period, only several patients are sufficient enough to map a gene responsible to a genetic disease. In fact, we applied this approach and successfully mapped the loci and later identified genes responsible for Fukuyama muscular dystrophy^23,24)^ and gelatinous drop-like corneal dystrophy.^25,26)^

Another advantage of microsatellite markers is for transmission disequilibrium test (TDT) to search genetic loci associated with common diseases. One example of the successes through this approach was shown by Onouchi *et al.*^27)^ using 78 Japanese sib pairs with Kawasaki disease; we demonstrated that an SNP in the ITPKC gene was the risk factor for the disease and associated with response to the intravenous immunoglobulin therapy.

## 4 SNP and international HapMap project

Around year 2000 when we expected the completion of human genome sequences, many scientists planned to construct a high-density single nucleotide polymorphism (SNP) map with millions of SNP markers covering an entire genome. SNPs are the most common genetic variations (one in 300 base pairs or less) in our genome. On the basis of the hypothesis of “common variant–common disease” association, SNPs were expected to be extremely informative as a tool for population genetics to identify genes (genetic variations) susceptible to various common diseases.

In Japan, the Japanese government announced a 5-year “Japanese Millennium Project” in 2000, which included the construction of Japanese SNP database and establishment of a high-throughput SNP analysis system for genome-wide association study. We have re-sequenced two dozens of Japanese individuals in a total of approximately 154-Mb of exonic segments and found more than 200,000 genetic variations. In addition, we established a high-throughput SNP genotyping system by a combination of multiplex PCR reactions (simultaneous amplification of 96 genomic segments) with Invader assay method. We then constructed the JSNP database including the information of nearly 190,000 SNPs and their allelic frequencies in 768 Japanese individuals in 2002 (the largest SNP database at that time).^28)^

Since multiple large-scale SNP genotyping platforms were developed around 2003, construction of the international consortium for making the SNP (Haplotype) database for three populations had been discussed and finally the international HapMap project including six countries (Canada, China, Japan, Nigeria, U.K., and U.S.A.) was organized in 2003. The aims of this consortium were clearly described in a paper published in Nature in 2003 as follows; “The goal of the International HapMap Project is to determine the common patterns of DNA sequence variation in the human genome and to make this information freely available in the public domain. An international consortium is developing a map of these patterns across the genome by determining the genotypes of one million or more sequence variants, their frequencies and the degree of association between them, in DNA samples from populations with ancestry from parts of Africa, Asia and Europe. The HapMap will allow the discovery of sequence variants that affect common disease, will facilitate development of diagnostic tools and will enhance our ability to choose targets for therapeutic intervention”.^29)^

After the very extensive efforts of participating scientists, the consortium constructed a database consisting of more than one million SNPs in 2005^30)^ and reported an extended set in 2007.^31)^ Although the majority of genetic variations were commonly shared among three major populations, a small subset of variations was detected in one particular population. Among them, an SNP in the ABCC11 gene that was uniquely found in the Asian population was later shown to be a determinant of human earwax type.^32)^ This kind of data suggested that if they have any effects on the quality or quantity of the gene product, which evolutionally fits to the certain environmental conditions, SNPs uniquely observed in a certain population might be determinants to characterize them.

## 5 Biobanks and GWAS

Although there were many criticisms and skepticisms for the “common variation-common diseases” approach for identifying genes (genetic variants) susceptible to common diseases at the beginning of the international HapMap project, many published papers have demonstrated the usefulness of genome wide association study (GWAS) to uncover various genetic factors associated with various diseases including “uncommon diseases”. Although not widely recognized, we started in 2001 whole-genome association studies using nearly 90,000 SNPs that well covered regions containing genes and identified a gene susceptible to myocardial infarction,^33)^ which is the first successful GWAS report in the world.^34)^ We also begun a BioBank Japan project in 2003 and have so far collected DNA samples from nearly 440,000 cases having either of 51 diseases. In combination with advanced technologies and the BioBank Japan samples, we have reported many candidate genes/loci possibly associated with susceptibility to diseases as summarized in Table 1. In other countries, systematic GWAS studies started in 2005 after the international HapMap project offered a large set of SNP information with the development of cheap, commercially-available, and accurate high-throughput SNP typing platforms. These technical and scientific advancement made GWAS a genetic analysis tool in the world, and the number of published GWAS reports exceeded more than 500 by the end of 2009 and reached 3567 in 2018.^35)^

After the initial wave of GWAS, the importance of bigger genetic data was soon recognized, and the international collaborative consortia were organized to combine internationally existing samples. Because linkage disequilibrium (LD) structures are different among populations, this kind of meta-analysis can be simply done only if the ethnicity is similar among the participated groups. However, more sophisticated analysis to combine trans-ancestry samples could contribute to an increase of the statistical power for discovery and finer causative variant mapping.^36)^ For example, trans-ancestry analyses conducted by AFGen consortium for atrial fibrillation^37)^ or MEGASTROKE consortium for stroke^38)^ took this strategy, and identified 97 atrial fibrillation loci or 32 stroke loci, respectively. The BioBank Japan project substantially contributed to both studies as the largest single non-European sample set.

These efforts aggregately led to the great accomplishments for understanding the complex disease-related genomics. Firstly, GWAS identified more than hundreds or thousands of susceptibility loci; for example, we found 1,407 loci for medical tests^39)^ and 276 loci for diseases^40)^ using BioBank Japan data set, which has supported the validity of polygenic model hypothesis proposed about 100 years ago.^41)^ Secondly, many polygenic susceptibility variants were shown to be enriched for non-coding regulatory DNA elements,^42–44)^ along with experimental evidence showing that GWAS variants alter the regulatory role of enhancers,^45,46)^ indicating that the genetics of complex traits is mainly governed by the diversity in gene regulation system among different individuals. This further enables cell-type specificity analysis using only genomic data; we found the enrichment of obesity-related loci in a B-cell specific active enhancer region.^47)^ Lastly, although the effect sizes of individual variants are modest or small, the cumulative effect of thousands of variants (polygenic score; PGS) was shown to have the predictive ability equivalent to a monogenic driver variant.^48)^

However, there is a serious bottleneck in utilizing GWAS results into clinic. PGS will not predict so accurately when the ethnicities of target populations used for GWAS are different.^49)^ This predictivity bias may happen even when the geographical regions within Japan are different.^50,51)^ The fact that ∼79% of GWAS participants are from European ancestry while they make up only 16% of the global population hampers the clinical utility of PGS. To alleviate this Eurocentric bias, non-European large genomic data sets are imperative. Ongoing world-wide efforts including the BioBank Japan and other biobanks are trying to address this problem (see Koyama *et al.*^52)^ for example).

## 6 Pharmacogenomics

It is now widely recognized that germline variations/somatic mutations in genes involved in drug metabolisms, drug transports, drug receptors, and drug target/downstream molecules, influence the effectiveness and adverse reaction risks of various drugs.

Adverse drug reactions (ADRs) can be classified simply into two groups, one that can be explained by the mode of therapeutic drug actions and the other for which little or no information was available for their underlying mechanisms. Typical examples in the former cases are leukocytopenia, nausea/vomiting, and hair loss caused by cytotoxic anti-cancer drugs as well as bleeding in brain or intestines caused by anticoagulants. Representative examples for the latter one are severe dug eruption and drug-induced liver injury caused by various drugs although risk factors for some of them have been identified in the last two decades. Millions of patients suffer from severe ADRs worldwide and a small subset of patients lose their lives. Since genetic variations are known to be one of the important factors among multiple factors involved in the etiology of ADRs, we are able to apply such genetic factors that increase risks of ADRs to improve medical management for patients having the high risk for ADRs. The improved management should also contribute to significant reduction of the unnecessary medical costs (one example for warfarin in https://core.ac.uk/display/6665518).

In the last decade, extensive research challenges have been taken to uncover genetic factors underlying ADRs and have successfully identified the genetic variations that increase risks of ADRs. Genetic biomarkers that have been so far clarified are listed in “Table of Valid Genomic Biomarkers in the Context of Approved Drug Labels” in U.S. Food and Drug Administration homepage (https://www.fda.gov/drugs/science-and-research-drugs/table-pharmacogenomic-biomarkers-drug-labeling). Proteins encoded by listed genes in the websites are classified mainly into three major groups.

Group 1: Drug metabolizing enzymes and drug transporters related to pharmacokinetics.

Group 2: Proteins related to pharmacodynamics.

Group 3: Human leukocyte antigens (HLAs).

It is obvious that poor clearance of drugs from our body results in elevation of drug concentration, leading to the toxic events; for example, genetic variants in UDP glucuronosyltransferase 1A1 (UGT1A1) that metabolizes irinotecan were shown to an association with myelotoxicity.^53)^ Similarly, inactivating variants of CYP2C9, which metabolizes warfarin, increase the risk of bleeding.^54)^ Furthermore, as an example of group 2, genetic variations in VKORC1 involved in vitamin K recycling influence pharmacodynamics of warfarin and define the necessary dose of warfarin in individual patients.^54)^

For life-threatening drug skin adverse reactions of unknown causes, drug-HLA interactions have been proven to be triggers for severe immune reactions attacking to skin cells; firstly, an HLA-B*1502 allele was shown to be very strongly linked to serious dermatologic reactions such as toxic epidermal necrolysis (TEN) and Stevens-Johnson syndrome (SJS) in patients taking carbamazepine.^55)^ Following their discovery, we found a strong association of an HLA-B*3505 allele with nevirapine-induced skin adverse drug reactions in Thai patients.^56)^ Although the exact molecular mechanisms how HLA molecules can be a trigger to induce such serious immune reactions are unclear, we and others suspect that a certain drug (or drug metabolite) binds to a certain HLA (or an HLA-peptide complex), is recognized as the presence of a non-self molecule, activates cytotoxic CD8^+^ T cells that can recognize the HLA-antigen complex, and then cause deleterious reactions.

In addition, some cases that influence the effectiveness of drugs were reported. A good example (although still in a big debate) is genotypes of cytochrome P450 2D6 (CYP2D6) and clinical outcome of breast cancer patients treated with tamoxifen. It is in an agreement that CYP2D6 metabolizes tamoxifen to its anti-estrogenic metabolites, 4-hydroxytamoxifen and endoxifen, and patients with some CYP2D6 variants show the significantly lower plasma concentration of endoxifen. Higher incidences of recurrence after breast cancer surgical treatment were observed in patients with null or low-activity genotypes in Caucasian and Japanese populations when patients received the tamoxifen monotherapy.^57,58)^ However, some groups, which included the cases treated with hormone therapy in combination of chemotherapy, insisted to conclude no association between CYP2D6 genotypes and the outcome of tamoxifen treatment.^59)^ Since patients need to take tamoxifen for 5–10 years, we are confident that the genetic diagnosis for tamoxifen treatment should improve the prognosis of breast cancer patients.

Although a large amount of information in the pharmacogenomics field has been accumulated, it is still limited in effective uses in clinical setting. Avoidance of ADRs certainly makes the quality of patient’s quality of life better and reduce the unnecessary medical costs in any countries. We need to construct an international network to further accelerate the application of useful pharmacogenomics information.

## 7 Immunogenomics and immunopharmacogenomics

As mentioned above, pharmacogenomics is a research area that combine pharmacology with genomics to examine the role of genetic variations (including somatic mutations) in drug responses. “Immunogenomics” is defined as a research filed which examines genetic variations in human leukocyte antigen (HLA) molecules or expression levels of immune-related genes in certain disease conditions. Due to the very rapid advancement in genomic analysis, we have been proposing the research area of “immunopharmacogenomics (IPG)” which further investigate the detailed immune responses including immuno repertoire changes at genomic levels in combination with pharmacological responses, as defined in a book entitled “Immunopharmacogenomics”.^60)^ Particularly, T and B lymphocytes, which have receptors, the T cell receptor (TCR) and B cell receptor (BCR), respectively, are well known to play essential roles in the adaptive immune system and are critically important for inducing the various immunological reactions. T and B lymphocytes need to prepare for responding to exposures to pathogens and chemicals with a huge diversity. Considering the extremely-high complexity of the immune cells and responses, a comprehensive approach to fully characterize chronological changes (also may be at anatomical locations) of the repertoires of T-cell and B-cell receptors according to the disease progression/remission are urgently and essentially required for better understanding of roles of immune cells and their chronological changes in drug responses as well as various disease or allergic conditions including anaphylaxis caused by vaccinations.

During the differentiation of lymphocytes, the genes encoding TCRs and BCRs, which include variable (V), diversity (D) (not for TCR α), and joining (J) exon segments, need to go through the essential biological process called “rearrangement” to generate functional receptors for huge numbers of pathogens. Extremely-high diversity of TCR and BCR repertories can be generated through combinations of each of V, (D), and J exon segments with dozens of distinct segments as well as through the template-independent insertion and deletion of nucleotides at rearranged junctions. The high variable complementary determining region 3 (CDR3) regions, which are critically important to determine the specificity and affinity for the antigen recognition by T cells, was generated by the combination of V-(D)-J rearrangement and insertion/deletion at the junction sites. The complexity of the TCR repertoire is still not fully clarified, but it is estimated to be approximately 10^15–18^ as a heterodimer of the TCR α and β chains in human. In addition, the higher diversity of the BCR repertoire is also well known because of the much higher somatic hyper-mutation processes in the BCR gene.

**Figure 1.**
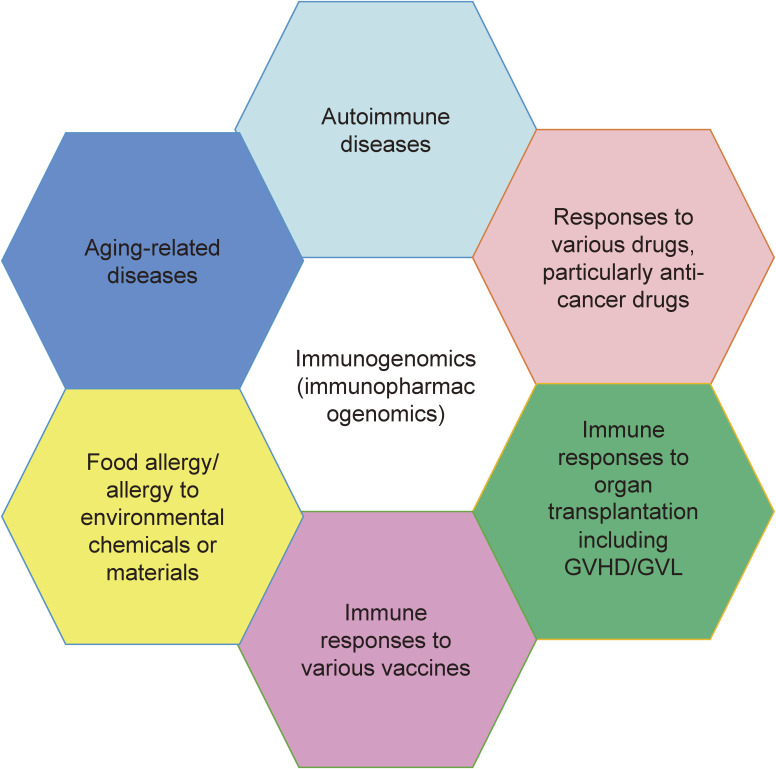
Importance of immunogenomics/immunopharmacogenomics in various medical research areas.

Considering the importance of immunogenomics analysis in coming years, we developed a systematic, accurate and unbiased analysis of TCR and BCR transcripts using the next generation sequencing technology and proposing their application in medical research areas as shown in Fig. 1. Firstly, further genome-wide and immunogenomics analyses of drug-induced adverse reactions (skin hypersensitivity and liver injuries) can find the risk genetic factors to cause these reactions. In fact, we reported the two genetic factors to increase the risk of severe skin hypersensitivity; an HLA-B*3505 allele for nevirapine-induced skin adverse drug reactions in Thai HIV-infected patients^56)^ as well as an HLA-A*3101 allele for carbamazepine-induced skin adverse drug reactions in Japanese population.^61)^ Drug-induced liver injuries were also indicated their associations with HLA molecules.^62)^ Secondly, the immunorepertoire analysis may help better understanding of the acute and chronic graft-versus-host disease (GVHD) and graft-versus-leukemia (GVL) effect of bone marrow (or cord blood) transplantation or tissue rejection after organ transplantation, leading to development of novel therapies. We reported that T cell repertoire analysis may predict the occurrence of GVHD in weeks earlier than when patients show the clinical symptoms.^63)^ Thirdly, it is obvious that we can analyze how individual immune cells play key roles in autoimmune diseases and food allergy (or allergy to chemicals). We have analyzed the BCR repertoires in Kawasaki disease patients before and after globulin treatment^64)^ as well as peanut allergy patients who received de-sensitization,^65)^ and found the drastic changes of BCR repertoires during the treatment. Fourthly, immunogenomics analysis (immune signature analysis) in combination with somatic mutation analysis in tumors may predict the efficacy of not only immunotherapy but also chemotherapy or radiation therapy.^66)^ In addition, through the BCR analysis we interestingly identified clonal expansion of plasma cells in a pseudo-progression tumor in a patient who received the immune checkpoint inhibitors.^67)^ Fifthly, immune repertoire drastically changes during the aging and should be important to understand various aging-related changes such as the severity of COVID-19 infection. Lastly, it is undoubtedly essential for investigating immune responses after vaccination, particularly anaphylactic reactions that occurred immediately after the vaccination.

## 8 Precision medicine

In 2015, Barack Obama, the former president of U.S., announced a new research initiative called “Precision Medicine Initiative (PMI)”.^68)^ The main aim was to take individual differences into account to improve health; the concept of “an appropriate dose of a right drug to a right patient” was not new and had been called as “personalized medicine”. The increasing availability of the advanced technologies including genomics, proteomics, metabolomics and other areas will encourage the realization of not only cancer precision medicine, but also of the whole range of health and medical cares including prevention of most of life style-related diseases.

It is not surprising to think that one of the scientific bases of PMI should be the genetic variation-aware personalized health care, and the utilization of PGS should be the promising implementation. However, the lack of portability of PGS among different populations needs to be addressed first. At present, the implementation of PGS will offer a far better precision to the subjects from European ancestry than those from the other populations, but scientists in the world need to consider this issue seriously as we previously had the good spirit to deliver the SNP information to all three populations in the international HapMap project. There would be several possibilities to overcome it as the ongoing international efforts of whole genome sequencing of the world-wide population would be crucial to find out the clues and resolve this issue. We should explore the road to the sustainable implementation of precision medicine based on genetic variations to all the people on the earth.

## 9 Conclusion

We here described briefly the history of human genetic variations as well as their significance in life science, particularly in medical genetics, pharmacogenetics and immunogenomics. One of the major goals in life science is to improve our quality of lives anywhere in the world. We believe that understanding of the molecular mechanisms causing diseases is essential and critical for the achievement of our goals. Genetic variations have played and will play very important roles in our medical and health cares. However, we must stress that we should pay much more efforts to avoid genetic discrimination. We need to teach that as genetic variations tell us, we are different, but we are equal and should respect the differences each other.

## Figures and Tables

**Table 1.** Genes susceptible to various diseases reported from our centers (Center for Genomic Medicine/Integrative Medical Sciences, RIKEN and Human Genome Center, Institute of Medical Science/Graduate School of Frontier Sciences, The University of Tokyo)

Trait	Association	Reference
**Adolescent idiopathic scoliosis**		
	*LBX1*	Takahashi. Nat. Genet. 2011;43(12):1237–1240.
	*GPR126*	Kou. Nat. Genet. 2013;45(6):676–679.
	*BNC2*	Ogura. Am. J. Hum. Genet. 2015;97(2):337–342.
**Age-related macular degeneration**		
	*TNFRSF10A*, 4q12	Arakawa. Nat. Genet. 2011;43(10):1001–1004.
**Atopic dermatitis**		
	8 new loci	Hirota. Nat. Genet. 2012;44(11):1222–1226.
**Atrial fibrillation**		
	6 new loci	Low. Nat. Genet. 2017;49(6):953–958.
**Body mass index**		
	*CDKAL1*,*KLF9*	Okada. Nat. Genet. 2012;44(3):302–306.
	112 new loci	Akiyama. Nat. Genet. 2017;49(10):1458–1467.
**Bronchial asthma**		
	3 new loci	Hirota. Nat. Genet. 2011;43(9):893–896.
	*WBSCR28*, *LRRC3B-NEK10*	Ishigaki. Nat. Genet. 2020;52(7):669–679.
**Cataract**		
	*FLRT2*	Ishigaki. Nat. Genet. 2020;52(7):669–679.
**Cerebral aneurysm**		
	*SIRT3*, *AEBP2-PDE3A*	Ishigaki. Nat. Genet. 2020;52(7):669–679.
**Chronic kidney disease**		
	17 new loci	Okada. Nat. Genet. 2012;44(8):904–909.
**Chronic obstructive pulmonary disease (COPD)**		
	*ACAD10*	Ishigaki. Nat. Genet. 2020;52(7):669–679.
**Duodenal ulcer**		
	*PSCA*,*ABO*	Tanikawa. Nat. Genet. 2012;44(4):430–434, S1–S2.
**Endometriosis**		
	*CDKN2BAS*	Uno. Nat. Genet. 2010;42(8):707–710.
**Gastric cancer**		
	*GUCY2F-IRS4*	Ishigaki. Nat. Genet. 2020;52(7):669–679.
**Graves’ disease**		
	3 new loci	Ishigaki. Nat. Genet. 2020;52(7):669–679.
**HCV-positive hepatocellular carcinoma**		
	*MICA*	Kumar. Nat. Genet. 2011;43(5):455–458.
**Hepatitis B**		
	*HLADP*	Kamatani. Nat. Genet. 2009;41(5):591–595.
**Hepatocellular carcinoma**		
	*DEPDC5*	Miki. Nat. Genet. 2011;43(8):797–800.
	*IFNL2-IFNL3*	Ishigaki. Nat. Genet. 2020;52(7):669–679.
**Interstitial lung disease**		
	*TMEM38B-ZNF462*	Ishigaki. Nat. Genet. 2020;52(7):669–679.
**Kawasaki disease**		
	*ITPKC*	Onouchi. Nat. Genet. 2008;40(1):35–42.
	3 new loci	Onouchi. Nat. Genet. 2012;44(5):517–521.
**Keloid**		
	1q41, *FOXL2*, *NEDD4*	Nakashima. Nat. Genet. 2010;42(9):751–754.
	*PHLDA3*	Ishigaki. Nat. Genet. 2020;52(7):669–679.
**Osteoarthritis**		
	*GDF5*	Miyamoto. Nat. Genet. 2007;39(4):529–533.
	*DVWA*	Miyamoto. Nat. Genet. 2008;40(8):994–998.
**Lumbar disc herniation**		
	*CILP*	Seki. Nat. Genet. 2005;37(6):607–612.
	*COL11A1*	Mio. Am. J. Hum. Genet. 2007;81(6):1271–1277.
	*THBS2*	Hirose. Am. J. Hum. Genet. 2008;82(5):1122–1129.
**Lung adenocarcinoma**		
	*TERT*, *TP63*	Miki. Nat. Genet. 2010;42(10):893–896.
**Lung cancer**		
	*POT1*	Ishigaki. Nat. Genet. 2020;52(7):669–679.
**Myocardial infarction/Coronary artery disease**		
	*LTA*	Ozaki. Nat. Genet. 2002;32(4):650–654.
	*LGALS2*	Ozaki. Nature. 2004;429(6987):72–75.
	*PSMA6*	Ozaki. Nat. Genet. 2006;38(8):921–925.
	*BRAP*	Ozaki. Nat. Genet. 2009;41(3):329–333.
	35 new loci	Koyama. Nat. Genet. 2020;52(11):1169–1177.
**Ossification of the posterior longitudinal ligament of the spine (OPLL)**		
	5 new loci	Nakajima. Nat. Genet. 2014;46(9):1012–1016.
**Osteoporosis**		
	*STK39*	Ishigaki. Nat. Genet. 2020;52(7):669–679.
**Prostate cancer**		
	5p15, *GPRC6A-RFX6*, 13q22, *FOXP4*	Takata. Nat. Genet. 2010;42(9):751–754.
	11q12, 10q26, 3p11.2	Akamatsu. Nat. Genet. 2012;44(4):426–429, S1.
	12 new loci	Takata. Nat. Commun. 2019;10(1):4422.
**Rheumatoid arthritis**		
	*PADI4*	Suzuki. Nat. Genet. 2003;34(4):395–402.
	*SLC22A4*	Tokuhiro. Nat. Genet. 2003;35(4):341–348.
	*FCRL3*	Kochi. Nat. Genet. 2005;37(5):478–485.
	*CD244*	Suzuki. Nat. Genet. 2008;40(10):1224–1229.
	*CCR6*	Kochi. Nat. Genet. 2010;42(6):515–519.
	9 new loci	Okada. Nat. Genet. 2012;44(5):511–516.
	42 new loci	Okada. Nature. 2014;506(7488):376–381.
**Stroke**		
	*PRKCH*	Kubo. Nat. Genet. 2007;39(2):212–217.
**Type 2 diabetes**		
	*WNT5B*	Kanazawa. Am. J. Hum. Genet. 2004;75(5):832–843.
	*KCNQ1*	Unoki. Nat. Genet. 2008;40(9):1098–1102.
	7 new loci	Imamura. Nat. Commun. 2016;7(1):10531.
	28 new loci	Suzuki. Nat. Genet. 2019;51(3):379–386.
	7 new loci	Ishigaki. Nat. Genet. 2020;52(7):669–679.
**Ulcerative colitis**		
	*FCGR2A*, 13q12, *SLC26A3*	Asano. Nat. Genet. 2009;41(12):1325–1329.
**Urolithiasis**		
	*STIM1*	Ishigaki. Nat. Genet. 2020;52(7):669–679.
**Uterine fibrosis**		
	3 new loci	Cha. Nat. Genet. 2011;43(5):447–450.

## Abbreviations

RFLP: restriction fragment length polymorphism
VNTR: variable number of tandem repeat
STR: short tandem repeat or microsatellite
SNP: single nucleotide polymorphism
CNV: copy number variation
SV: structural variation
PCR: polymerase chain reaction
